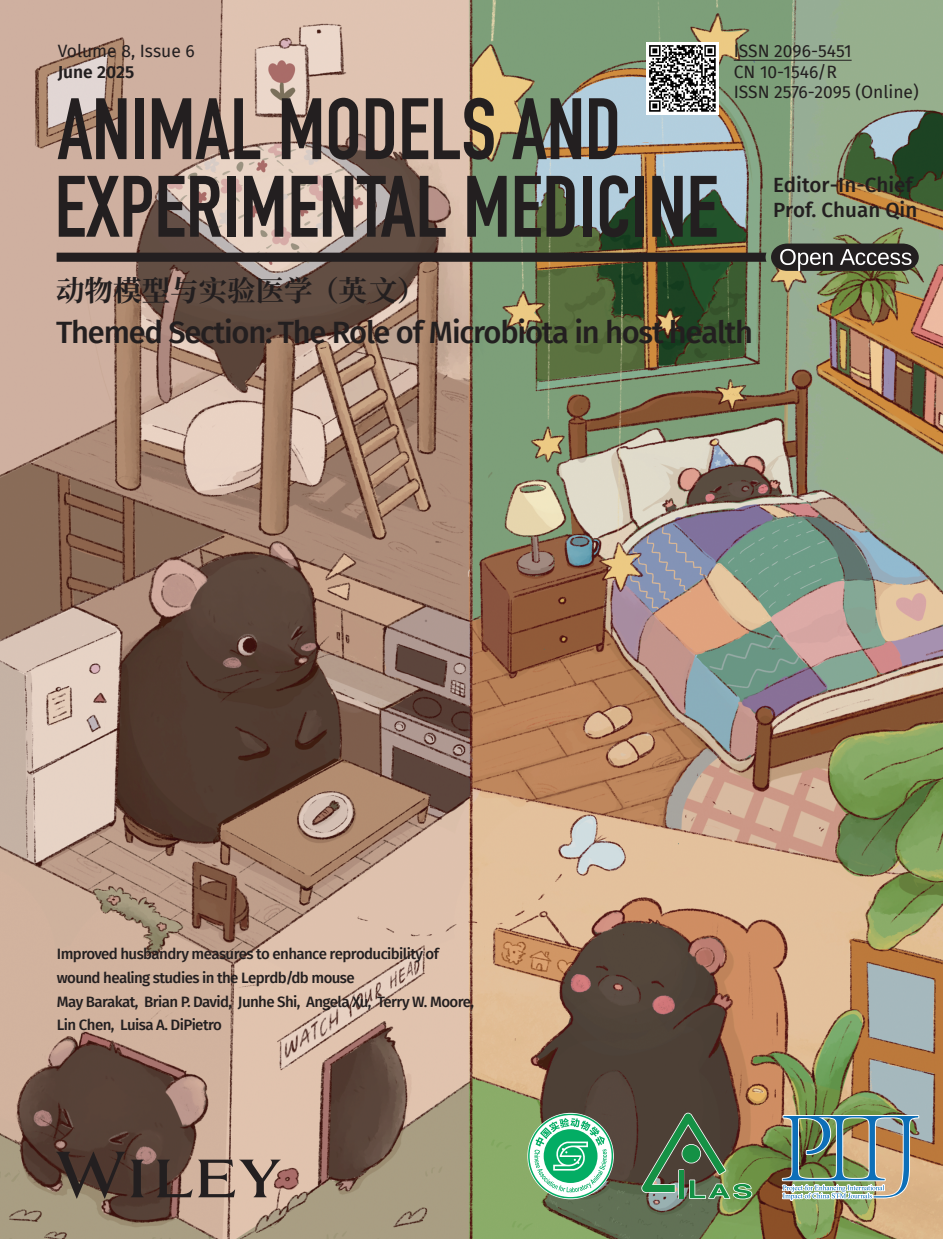# Cover Picture

**DOI:** 10.1002/ame2.12432

**Published:** 2025-06-27

## Abstract

This cover image is based on the article “Improved husbandry measures to enhance reproducibility of wound healing studies in the Leprdb/db mouse” reported by May Barakat, Brian P. David, Junhe Shi, Angela Xu, Terry W. Moore, Lin Chen, Luisa A. DiPietro. (https://doi.org/10.1002/ame2.70010). To highlight the impact of improved care and husbandry of the db/db mice, we designed the cover with a split depiction of the mice: first, living in a smaller, uncomfortable home that clearly doesn't accommodate their size or needs, and second, living comfortably in a personalized space that suits them perfectly. We hope this artwork will not only emphasize the importance of improved care for experimental animals but also inspire some joy and appreciation for these special mice and their unique qualities.